# A 30-year analysis of urolithiasis burden in the North Africa and Middle East region: Findings from the global burden of disease study 2021

**DOI:** 10.1371/journal.pone.0343341

**Published:** 2026-05-04

**Authors:** Sina Golestani, Mohammad Taghi Talebian, Ali Golestani, Zeinab Ahadi, Ozra Tabatabaei-Malazy, Ziba Aghsaei Fard, Abdolreza Mohammadi, Bagher Larijani, Seyed Mohammad Kazem Aghamir

**Affiliations:** 1 Non-Communicable Diseases Research Center, Endocrinology and Metabolism Population Sciences Institute, Tehran University of Medical Sciences, Tehran, Iran; 2 Sina Hosspital, Tehran University of Medical Sciences, Tehran, Iran; 3 Urology Research Center, Tehran University of Medical Sciences, Tehran, Iran; 4 Endocrinology and Metabolism Research Center, Endocrinology and Metabolism Clinical Sciences Institute, Tehran University of Medical Sciences, Tehran, Iran; Metrohealth Medical Center, UNITED STATES OF AMERICA

## Abstract

**Background:**

Urolithiasis, a prevalent urological disorder, is associated with significant morbidity and economic burden. Despite the Global Burden of Disease (GBD) data, regional specificity for urolithiasis burden in North Africa and Middle East (NAME) remains limited. This study aims to fill this gap by analyzing the burden of urolithiasis in the NAME region from 1990 to 2021.

**Methods:**

Data from the GBD 2021 study were used to evaluate key health measures, including incidence, prevalence, mortality, years lived with disability (YLDs), years of life lost (YLLs), and disability-adjusted life years (DALYs). Age-standardized rates (ASRs) and absolute numbers were assessed across 21 NAME countries, stratified by sex, age, and sociodemographic index (SDI). Results were presented with 95% uncertainty intervals.

**Results:**

In 2021, the overall incidence reached 5.3 million (95% uncertainty intervals: 4.2–6.8) cases, compared to 2.0 million (1.6–2.5) in 1990. Prevalence rose from about 76,000 (61,000–96,000) cases in 1990–201,000 (160,000–257,000) in 2021. The number of deaths increased considerably from 142.1 (79.8–194.5) to 394.2 (182.4–509.5), and the DALYs rose from 10,814.0 (7,970.0–13,932.3) to 25,213.3 (17,943.5–33,787.7) from 1990 to 2021. ASRs for all burden measures remained stable and females consistently exhibited lower rates compared to males. There was a positive correlation between SDI and rates of incidence, prevalence, and YLDs; however, deaths, YLLs, and DALYs exhibited no significant correlation with SDI.

**Conclusion:**

Urolithiasis imposes a growing health and economic burden in the NAME region, particularly among middle-aged populations and high-SDI countries. Targeted interventions and region-specific policies are crucial to address the rising disease burden effectively.

## Introduction

Urolithiasis is a common urological disorder characterized by the formation of stones or calculi in the kidneys, bladder, or urethra. These stones can vary in composition, with calcium oxalate being the most prevalent type, accounting for a significant proportion of cases [1]. Clinically, urolithiasis is associated with a range of medical complications, including renal colic and hydronephrosis. Bilateral lithiasis, which more likely affects males and causes hyperuricemia, presents greater clinical challenges than unilateral stones. Additionally, the disorder has a high recurrence rate, which imposes substantial social and economic burdens on individuals and healthcare systems [2,3]. The etiology of urolithiasis is multifactorial, involving a complex interplay of genetic, environmental, and lifestyle factors. Several risk factors contribute to its development, such as dietary patterns, insufficient water intake, alcohol consumption, high body mass index (BMI), sedentary lifestyles, and excessive consumption of red meat [4–6]. Despite its health impact, urolithiasis awareness remains low in North Africa and Middle East (NAME) countries like Saudi Arabia [7]. This underscores the importance of raising awareness and understanding this condition to mitigate its impact.

Globally, urolithiasis exhibits a prevalence rate ranging from 1% to 20% across different regions [8]. It is more commonly diagnosed in males than females, reflecting a sex disparity in its occurrence [9]. While the absolute numbers of cases, as well as disability-adjusted life years (DALYs) attributable to urolithiasis, have been increasing, age-standardized rates (ASRs) of its global burden have shown a decline. Interestingly, mortality rates associated with urolithiasis have remained stable over the years, though significant regional variability exists [10]. Regional studies present contrasting patterns, with the NAME region experiencing an 83.2% increase in urolithiasis cases between 2000–2021 [10], while Afghanistan in the Middle East demonstrated one of the world’s most substantial declines in incidence rates [11]. Cross-national comparisons reveal interesting variations, such as Iran’s higher urolithiasis prevalence compared to Jordan in the Middle Ease, potentially associated with differences in antibiotic prescribing practices [12]. The NAME region exhibits unique risk factors: elevated ambient temperatures [13,14] and excessive sodium consumption (among the highest globally), which promote hypercalciuria and lithogenesis [15,16]. The economic toll of this condition is considerable; in 2007, the total cost of managing urolithiasis was estimated at $3.79 billion in the United States, and this figure is projected to rise by an additional $780 million by 2030 [17]. Importantly, these costs may be mitigated through preventive measures, with increased water intake demonstrating particular efficacy as a cost-effective intervention [18]. These statistics highlight the pressing need for a deeper understanding of the disease burden in NAME region to develop cost-effective interventions and policies.

Given its high prevalence and significant economic impact, urolithiasis demands greater attention from policymakers and healthcare providers. A nuanced understanding of its burden, particularly its variability across different regions, is essential for effective resource allocation. Although global burden of disease (GBD) studies provide comprehensive estimates of disease burden, including urolithiasis, they often focus on global or national levels, with limited regional specificity [8–10,9–24]. To date, no study has specifically examined the burden of urolithiasis in the NAME region, despite the unique sociodemographic and environmental characteristics of this area. Our study addresses this gap through NAME-specific analyses, extending beyond GBD parameters to investigate Socio-Demographic Index (SDI)-stratified trends not available in standard GBD reports. Furthermore, we extend beyond GBD parameters by investigating the underlying drivers of urolithiasis burden trends in the region.

This study seeks to address this gap by utilizing GBD data to estimate key health measures associated with the burden of urolithiasis in the NAME region from 1990 to 2021. These measures include prevalence, incidence, mortality, years lived with disability (YLDs), years of life lost (YLLs), and DALYs, both as all age numbers and ASRs. The study further stratifies findings by age, sex, and SDI, providing a comprehensive view of the disease burden and its relationship with various health indicators in this region.

## Materials and methods

### Data source

The GBD 2021 study provides comprehensive annual estimates from 1990 to 2021, encompassing 371 diseases and injuries across 204 countries and territories. Additionally, it includes subnational data from hospitals and claims for more than 20 countries, offering a detailed and extensive framework for assessing global health trends [25]. Key health measures, including incidence, prevalence, deaths, YLDs, YLLs, and DALYs related to urolithiasis, were extracted for the 21 NAME countries over the period from 1990 to 2021. Data retrieval was facilitated using publicly available resources, such as the GBD Result tool (available via: https://vizhub.healthdata.org/gbd-results/) and the Global Health Data Exchange (GHDx) results tool (available via: http://ghdx.healthdata.org/gbd-results-tool). All analytical steps in the GBD study adhered to the Guidelines for Accurate and Transparent Health Estimates Reporting (GATHER) [26]. This study focuses on the NAME region, which is one of seven super-regions defined by GBD 2021. The NAME region comprises 21 countries: Afghanistan, Algeria, Bahrain, Egypt, Iran, Iraq, Jordan, Kuwait, Lebanon, Libya, Morocco, Oman, Palestine, Qatar, Saudi Arabia, Sudan, Syria, Tunisia, Turkey, the United Arab Emirates, and Yemen. Notably, this differs from the World Bank’s Middle East and North Africa (MENA) grouping, which excludes Sudan and Turkey but includes Israel. We maintained the GBD’s NAME classification to ensure methodological consistency with our primary data source, thereby facilitating valid comparisons with other GBD research while still encompassing the principal nations of epidemiological relevance in this geographical area.Urolithiasis was defined as abnormal formation of crystalline masses along the urinary tract using the International Classification of Diseases with corresponding codes from the 10th Revision (ICD-10: N20–N23) [27]. This study includes data for both sexes and analyzes health measures at all ages, as well as using ASRs to allow for better comparability across populations. The age stratification followed a detailed structure, beginning with 2–4 years, and continuing in 5-year intervals from the age of 5 onward.

### YLDs, YLLs, DALYs, and SDI estimates

Our burden estimation employed standardized GBD metrics to quantify both fatal and non-fatal health impacts of urolithiasis. For Years Lived with Disability (YLDs), we applied disability severity weights to various clinical manifestations of urolithiasis, representing their relative health impact. These weights were multiplied by condition-specific prevalence rates across severity categories, yielding an aggregate measure of non-fatal burden. Years of Life Lost (YLLs) were derived by multiplying age-specific mortality counts by the corresponding remaining life expectancy based on global reference standards, thus quantifying premature mortality [25].

The composite Disability-Adjusted Life Year (DALY) metric was calculated by summing YLDs and YLLs, providing a comprehensive measure of total disease burden. To enable cross-population comparisons, we age-standardized all estimates using the GBD reference population [25].

The SDI is a composite indicator designed to reflect a country’s developmental status. It consists of the geometric mean of lag-distributed income per capita, mean years of education among adults, and the total fertility rate for females under 25 years of age [25]. The index ranges from 0 to 1, where a value of 1 represents the highest level of sociodemographic development.

### Data analysis

The analysis employed two primary modeling tools: spatiotemporal Gaussian process regression (ST-GPR) and Disease Modelling-Meta regression (DisMod-MR) [25]. The relationship between SDI and burden measures was assessed using locally weighted regression and scatterplot smoothing (LOESS) regression. Uncertainty intervals (UIs) at the 95% confidence level were generated for each measure based on 500 draws from the posterior distribution, derived from the 2.5th and 97.5th percentiles. Statistical significance was determined by the absence of overlap in the 95% UIs [25]. To adjust for differences in population sizes, the data were presented as rates per 100,000 individuals. Furthermore, the age distribution was standardized using the GBD global standard population structure to account for variations in population age demographics [27]. Data visualizations were created using Python programming language (version 3.12.4).

### Ethics approval and consent to participate

This research was conducted in accordance with the principles of the Declaration of Helsinki. The findings are derived from aggregated estimates provided by the Global Burden of Disease (GBD) 2021 study, which comply with all applicable guidelines and regulations. As the study used publicly available, de-identified, aggregated data and did not involve direct interaction with human participants, no informed consent was required. This study was approved by the Ethics Review Board of Sina Hospital, Tehran University of Medical Sciences (IR.TUMS.SINAHOSPITAL.REC.1403.080).

## Results

### Trends in all-age measures

**Fig 1:**
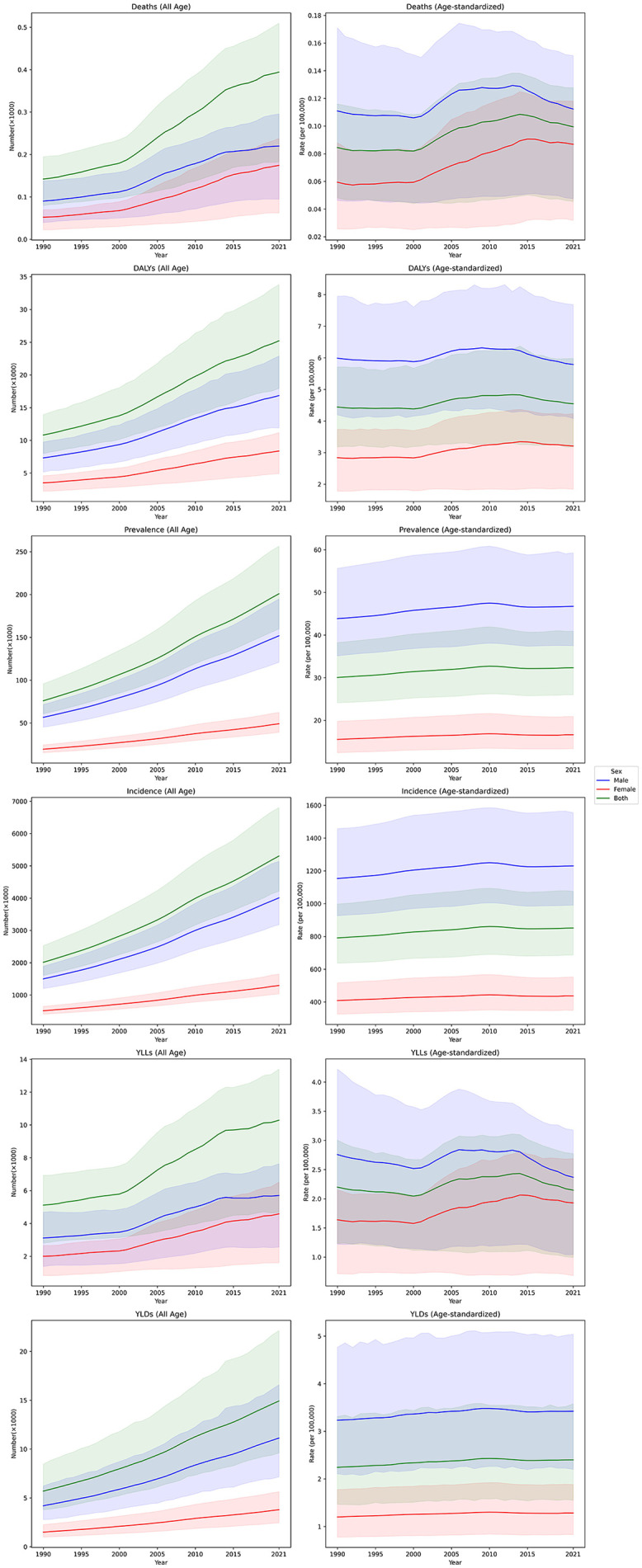
Trends in incidence, prevalence, deaths, YLDs, YLLs, and DALYs of urolithiasis from 1990 to 2021 across males, females, and both sexes. YLD = years lived with disability, YLL = years of life lost, DALY = disability-adjusted life years.

From 1990 to 2021, there was a marked increase in the burden of urolithiasis across multiple burden measures. This upward trend was observed for both males and females, although the numbers for females were consistently lower across all measures. Incidence rose significantly from 2.0 million (95% UI:1.6–2.5) in 1990 to 5.3 million (4.2–6.8) in 2021, by 163.4% (147.3–175.8). Similarly, the prevalence grew from 75,961.0 (60,829.6–95,790.6) to 200,852.9 (159,841.8–256,394.9) over the same period by 164.4% (148.6–176.7). The number of deaths attributed to urolithiasis also increased considerably, from 142.1 (79.8–194.5) in 1990 to 394.2 (182.4–509.5) in 2021. The DALYs associated with urolithiasis rose from 10,814.0 (7,970.0–13,932.3) in 1990–25,213.3 (17,943.5–33,787.7) in 2021, by 133.2% (93.0–175.4). While YLLs and YLDs number were approximately similar in 1990, YLD number was about 1.5 times of YLLs in 2021 (Fig 1 and Table 1).

**Table 1:** Comparison of urolithiasis incidence, prevalence, deaths, YLDs, YLLs, and DALYs in North Africa and Middle East region between 1990 and 2021.

Measure	Metric	Year						Percent change (1990–2021) (95% UI)		
		1990 (95% UI)			2021 (95% UI)					
		both	female	Male	both	female	male	both	female	male
**Deaths**	All ages Number	142.1 (79.8 to 194.5)	52.0 (22.7 to 69.8)	90.1 (39.5 to 138.1)	394.2 (182.4 to 509.5)	174.3 (62.5 to 238.0)	219.9 (94.8 to 295.6)	177.4 (63.0 to 289.1)	235.2 (88.9 to 412.8)	144.0 (36.5 to 283.3)
**Deaths**	ASR	0.1 (0.0 to 0.1)	0.1 (0.0 to 0.1)	0.1 (0.0 to 0.2)	0.1 (0.0 to 0.1)	0.1 (0.0 to 0.1)	0.1 (0.0 to 0.2)	17.7 (−32.9 to 63.6)	46.2 (−11.6 to 112.1)	1.2 (−45.7 to 57.9)
**DALYs**	All ages Number	10814.0 (7970.0 to 13932.3)	3497.1 (2231.5 to 4590.8)	7316.9 (5145.0 to 9753.6)	25213.3 (17943.5 to 33787.7)	8373.8 (4924.0 to 11174.9)	16839.5 (11909.5 to 22902.0)	133.2 (93.0 to 175.4)	139.4 (79.6 to 202.8)	130.1 (86.3 to 188.1)
**DALYs**	ASR	4.4 (3.2 to 5.7)	2.8 (1.8 to 3.7)	6.0 (4.2 to 7.9)	4.5 (3.2 to 6.0)	3.2 (1.8 to 4.2)	5.8 (4.1 to 7.7)	2.3 (−18.5 to 20.5)	13.1 (−19.0 to 39.2)	−3.3 (−23.5 to 18.8)
**YLDs**	All ages Number	5698.8 (3795.2 to 8459.9)	1497.2 (988.0 to 2258.4)	4201.5 (2782.4 to 6225.4)	14924.7 (9610.2 to 22123.7)	3791.5 (2451.0 to 5629.4)	11133.2 (7156.6 to 16581.4)	161.9 (142.5 to 184.6)	153.2 (136.9 to 166.9)	165.0 (140.2 to 193.8)
**YLDs**	ASR	2.2 (1.5 to 3.3)	1.2 (0.8 to 1.8)	3.2 (2.1 to 4.8)	2.4 (1.6 to 3.6)	1.3 (0.8 to 1.9)	3.4 (2.2 to 5.0)	7.0 (0.3 to 14.9)	6.9 (4.2 to 9.6)	5.8 (−2.9 to 16.4)
**YLLs**	All ages Number	5115.2 (2804.1 to 6931.6)	1999.9 (829.0 to 2660.8)	3115.3 (1377.9 to 4685.8)	10288.6 (4668.9 to 13397.8)	4582.3 (1601.2 to 6516.8)	5706.3 (2563.7 to 7633.8)	101.1 (17.6 to 188.7)	129.1 (26.3 to 282.1)	83.2 (8.0 to 214.6)
**YLLs**	ASR	2.2 (1.2 to 3.0)	1.6 (0.7 to 2.2)	2.8 (1.2 to 4.2)	2.1 (1.0 to 2.8)	1.9 (0.7 to 2.7)	2.4 (1.0 to 3.2)	−2.4 (−43.2 to 36.7)	17.6 (−36.0 to 75.8)	−14.1 (−51.8 to 35.6)
**Prevalence**	All ages Number	75961.0 (60829.6 to 95790.6)	19355.4 (15341.3 to 24386.2)	56605.6 (45153.0 to 71521.7)	200852.9 (159841.8 to 256394.9)	49077.9 (39124.9 to 62304.6)	151775.0 (120902.4 to 194794.8)	164.4 (148.6 to 176.7)	153.6 (137.3 to 167.3)	168.1 (152.2 to 180.4)
**Prevalence**	ASR	30.1 (24.1 to 38.2)	15.5 (12.4 to 19.8)	43.9 (35.1 to 55.6)	32.3 (26.0 to 40.9)	16.6 (13.4 to 20.9)	46.7 (37.5 to 59.3)	7.5 (6.0 to 9.3)	7.0 (4.7 to 9.3)	6.6 (4.6 to 8.8)
**Incidence**	All ages Number	2013198.6 (1612875.7 to 2532059.3)	512789.0 (407871.1 to 645938.6)	1500409.6 (1202259.7 to 1891919.6)	5303600.3 (4215645.1 to 6801403.6)	1295209.2 (1037493.5 to 1648394.8)	4008391.1 (3181258.7 to 5141710.1)	163.4 (147.3 to 175.8)	152.6 (135.9 to 166.3)	167.2 (150.3 to 180.1)
**Incidence**	ASR	791.1 (637.4 to 997.9)	408.6 (325.9 to 517.1)	1153.8 (926.7 to 1457.9)	851.4 (686.6 to 1075.2)	437.4 (348.9 to 552.8)	1230.4 (991.0 to 1555.4)	7.6 (6.2 to 9.4)	7.0 (4.8 to 9.3)	6.6 (4.8 to 8.8)

**• YLDs:** Years lived with disability; **YLLs:** Years of life lost; **DALYs:** Disability-adjusted life years; ASR = Age-standardized Rate.

### Trends in age-standardized rates

The ASRs of burden measures for urolithiasis showed no statistically significant change from 1990 to 2021. Across all measures, females consistently exhibited the lower rates compared to males. Except for the incidence ASR, which showed a slight increase of 7.6% (95% UI: 6.2–9.4) from 791.1 (637.4–997.9) per 100,000 population in 1990 to 851.4 (686.6–1,075.2) in 2021, other measures remained unchanged compared to 1990. In 2021, the prevalence ASR was 32.3 (26.0–40.9) per 100,000, the death ASR was 0.1 (0.0–0.1), and the DALYs ASR was 4.5 (3.2–6.0). The YLDs and YLLs ASRs were also similar, remaining approximately 2 per 100,000 population from 1990 to 2021 (Fig 1 and Table 1).

### Age-standardized rates in different countries

**Fig 2:**
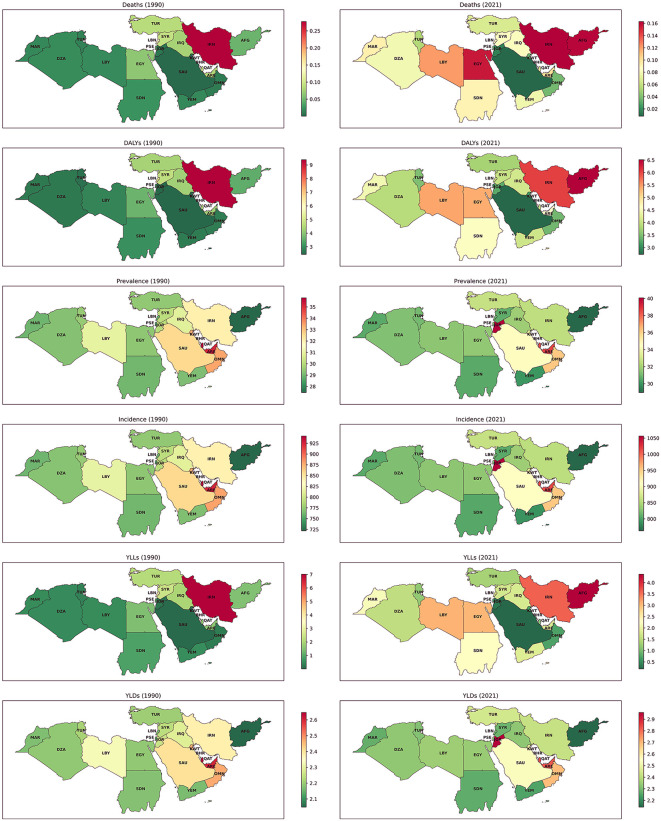
ASRs of incidence, prevalence, deaths, YLDs, YLLs, and DALYs for urolithiasis across NAME countries in 1990 and 2021. YLD = years lived with disability, YLL = years of life lost, DALY = disability-adjusted life years, ASR = Age-Standardized Rate.

**Fig 3:**
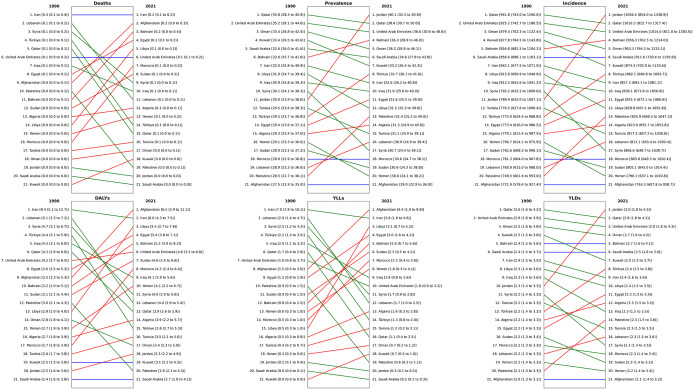
Rankings of ASRs for incidence, prevalence, deaths, YLDs, YLLs, and DALYs of urolithiasis in different NAME countries from 1990 to 2021. YLD = years lived with disability, YLL = years of life lost, DALY = disability-adjusted life years, ASR = Age-Standardized Rate.

In 1990, Qatar had the highest ASR of incidence at 941.8 (95% UI: 743.0–1206.0) per 100,000 population, whereas Afghanistan had the lowest rate, at 721.9 (578.4–927.4). Interestingly, when disaggregated by sex, Iran recorded the highest incidence rates for both males at 1223.0 (985.1–1555.7) and females at 438.7 (350.5–553.8) per 100,000 population. By 2021, the incidence rates increased in most countries, with Jordan emerging as the country with the highest rates at 1056.4 (854.0–1338.9) per 100,000, while Afghanistan continued to have the lowest rates, at 763.3 (607.8–958.7). A similar trend was observed in the ASR of prevalence. In 1990, Qatar led the region with the highest prevalence rate at 35.8 (95% UI: 28.3–45.9) per 100,000 population, while Afghanistan had the lowest prevalence at 27.5 (21.9–35.0). By 2021, prevalence rates increased across most countries in the region. Jordan recorded the highest prevalence at 40.1 (32.3–50.9) per 100,000, while Afghanistan remained at the lower end, with a rate of 29.0 (22.9–36.0) (Figs 2 and 3).

The ASRs of deaths and YLDs attributable to urolithiasis remained relatively low and stable across the NAME region in both 1990 and 2021, with death rates below 0.4 and YLDs ranging between 2 and 3 per 100,000 population. Regarding YLLs, Iran had the highest burden in 1990, at 7.0 (95% UI: 2.9–10.1) per 100,000. By 2021, Afghanistan recorded the highest YLLs, at 4.4 (1.0–8.9) per 100,000. The DALYs rates also highlighted disparities across countries. In 1990, Iran had the highest DALYs, at 9.4 (95% UI: 5.1–12.7) per 100,000, while Saudi Arabia reported the lowest at 2.4 (1.6–3.8). By 2021, Afghanistan had the highest DALYs, at 6.5 (2.9–11.1) per 100,000, whereas Saudi Arabia remained at the lower end with a rate of 2.7 (1.8–4.1) (Figs 2 and 3).

### Rates in different age groups

**Fig 4:**
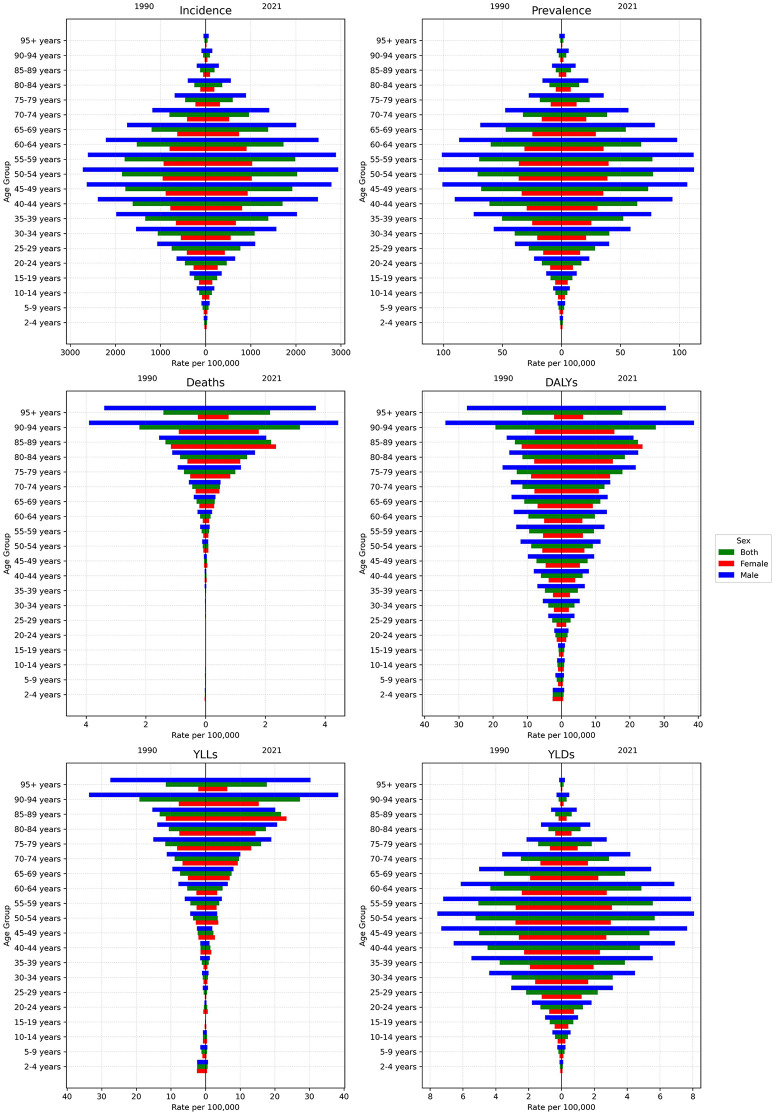
Rates of incidence, prevalence, deaths, YLDs, YLLs, and DALYs of urolithiasis across different age groups in 1990 and 2021. YLD = years lived with disability, YLL = years of life lost, DALY = disability-adjusted life years.

Fig 4 represents the comparison of age-specific rates per 100,000 population of burden measures related to urolithiasis across the NAME region in 1990 and 2021. The incidence, prevalence, and YLD rates exhibited a bell curve-like distribution across age groups, peaking at the 50–54-year age group for both males and females and showing consistent patterns over the years. Specifically, the incidence rate for the 50–54-year group increased from 1,854.0 (95% UI: 1,058.8–2,928.2) per 100,000 in 1990–2,031.7 (1,164.2–3,199.0) in 2021. Similarly, prevalence in the same age group rose from 71.0 (39.2–111.2) per 100,000 in 1990 to 77.6 (43.5–120.8) in 2021. For YLDs, the rate increased slightly, reaching 5.7 (2.7–10.1) per 100,000 in 2021 compared to 5.2 (2.5–8.9) in 1990 (Fig 4).

In contrast, deaths, YLLs, and DALYs were more pronounced in older age groups. The highest rates were observed in individuals aged 90–94 years for both sexes combined, while males and females individually peaked in the 90–94 and 85–89 age groups, respectively. In 1990, the death rate for the 90–94 age group across both sexes was 2.2 (95% UI: 0.9–3.9) per 100,000, which increased to 3.1 (1.4–4.4) in 2021. The YLL rate for this age group similarly rose, from 19.1 (7.7–33.7) per 100,000 in 1990 to 27.2 (11.9–37.7) in 2021. Finally, the DALY rate for individuals aged 90–94 years grew from 19.2 (7.8–33.8) per 100,000 in 1990 to 27.6 (12.3–38.0) in 2021. It is also noteworthy that while in 1990, the levels of DALYs and YLLs for age groups under 20 years old were minimal, they significantly decreased by 2021.

### Burden vs SDI

**Fig 5:**
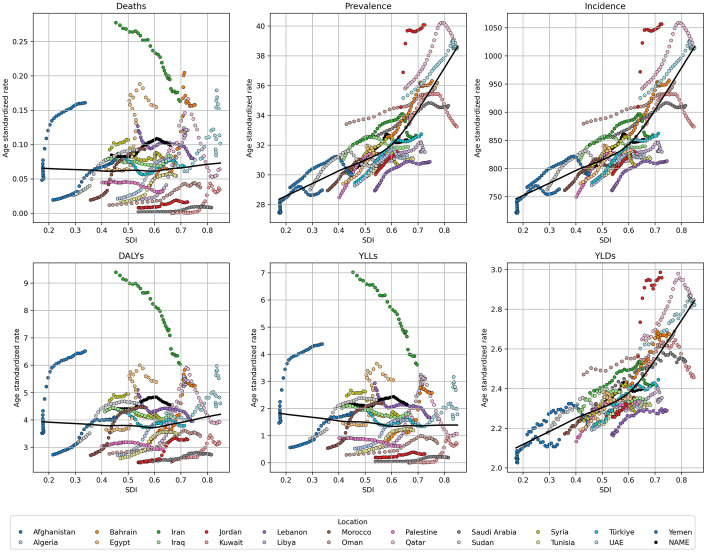
Relationship between SDI and ASRs of incidence, prevalence, deaths, YLDs, YLLs, and DALYs of urolithiasis in NAME countries (1990–2021). SDI = socio-demographic index, YLD = years lived with disability, YLL = years of life lost, DALY = disability-adjusted life years, ASR = Age-Standardized Rate.

Overall, the ASRs for incidence, prevalence, and YLDs exhibited a positive association with SDI. In contrast, deaths, YLLs, and DALYs demonstrated no significant correlation with SDI across the NAME region. Interestingly, some countries deviated from these overall trends and displayed unique patterns. For example, in Iran, a negative correlation was observed between SDI and deaths, YLLs, and DALYs (Fig 5).

## Discussion

This study provides a detailed evaluation of the burden of urolithiasis in the NAME region from 1990 to 2021, shedding light on key trends, disparities, and determinants. The findings revealed substantial increases in absolute numbers of incidence, prevalence, YLDs, and DALYs, underscoring the rising public health impact of urolithiasis, particularly among middle-aged populations. Interestingly, the ASRs for deaths and YLLs remained stable or declined slightly over time, suggesting improvements in healthcare access and disease management. Similar upward trends in the burden of urolithiasis have been observed in other global studies [28]. However, most studies indicate a decline in the burden from 1990 to 2019 in specific regions [9,21,22,29,30]. Additionally, evidence highlights that while some regions have experienced an increased burden from 2000 to 2021, others have shown a decline [10] emphasizing the need for region-specific analyses. The observed increase in urolithiasis burden within the NAME region likely results from multiple interacting factors, consistent with the disease’s multifactorial etiology. Existing literature implicates population aging, dietary pattern changes, and climate change impacts as potential contributors [31–35]. Additional environmental and metabolic determinants, which will be explored in subsequent sections, may further explain these epidemiological patterns.One of the most striking findings of this study is the heterogeneity in urolithiasis burden across the NAME region. High-SDI countries, such as Qatar, Iran, and Jordan, exhibited the highest rates of incidence and prevalence, while low-SDI countries, including Afghanistan and Sudan, consistently reported lower rates. This pattern aligns with other studies demonstrating a positive correlation between SDI and the burden of urolithiasis [22]. However, some studies have reported only a weakly positive correlation, indicating potential variability based on regional factors [9]. The observed disparities are likely influenced by differences in diagnostic capacity, healthcare access, and public awareness rather than true variations in prevalence. In high-SDI countries, the widespread availability of advanced imaging technologies, such as Computed Tomography (CT) scans, facilitates higher detection rates. Conversely, limited diagnostic resources in low-SDI settings may result in significant underreporting. Emerging technologies like augmented reality (AR) have shown promise in reducing the burden of urolithiasis in low- and middle-income countries, offering innovative pathways for improved diagnosis and management [36].

The positive association between SDI and non-fatal measures, such as incidence, prevalence, and YLDs, underscores the influence of socioeconomic development on the epidemiology of urolithiasis. While improved healthcare infrastructure and diagnostic capabilities in high-SDI countries contribute to higher detection rates, these regions also face lifestyle-related risk factors associated with development. Sedentary behavior, high BMI, and dietary patterns characterized by excessive intake of salt, sugar, and animal protein are prominent in high-SDI countries [37–39]. Such factors not only elevate the risk of urolithiasis but also exacerbate related conditions like hypertension, further increasing the disease burden [40]. Encouraging healthy dietary habits can mitigate these risks and reduce the prevalence of urolithiasis.

Age-specific analyses revealed distinct patterns, with the highest incidence, prevalence, and YLD rates occurring in the 50–54-year age group, consistent with the natural history of urolithiasis. This age group represents a peak period for stone formation due to metabolic and dietary factors. Similar trends have been reported in other studies, with peak rates observed in the 55–59 [22], 50–59 [41], and 30–49 age groups [42]. Conversely, deaths, YLLs, and DALYs were predominantly concentrated in older age groups, particularly those aged 85–94 years. This highlights the amplified impact of urolithiasis in the elderly, where comorbidities and decreased physiological resilience exacerbate disease outcomes.

Sex disparities were evident, with males consistently exhibiting higher rates of incidence, prevalence, and YLLs than females. These findings align with existing literature [43,44], and may be attributed to sex-specific risk factors such as hormonal differences, occupational exposures, and behavioral patterns. Testosterone, for instance, has been shown to inhibit osteopontin expression, a protective factor against urolithiasis, potentially explaining higher male susceptibility [45]. However, the faster rate of increase in urolithiasis burden among females observed in this study suggests that shifting lifestyle patterns, including dietary changes and reduced physical activity, may be narrowing the historical gender gap.

Environmental conditions in the NAME region significantly contribute to the high prevalence of urolithiasis. The arid climate and high temperatures characteristic of many countries in the region increase the risk of dehydration, a well-known contributor to urolithiasis [44,46,47]. Furthermore, the quality of drinking water in many parts of the NAME region may exacerbate this risk due to elevated concentrations of minerals which have been linked to an increased propensity for urinary crystallization [48,49]. Another emerging environmental concern is the rising level of air pollution, particularly fine particulate matter (PM2.5). Recent studies indicate that prolonged exposure to PM2.5, which is increasingly prevalent in urban areas of the NAME region [50], may elevate the risk of nephrolithiasis [51]. Additionally, food safety concerns related to heavy metal contamination—such as cadmium—have been identified in livestock and agricultural products across the Middle East [52]. Chronic ingestion of these toxic elements has been linked to stone formation [53,54]. Addressing these environmental determinants through public health initiatives, such as implementing water purification systems to reduce mineral and heavy metal content, alongside stricter regulations on industrial emissions and agricultural contaminants, could significantly reduce the disease burden.

Metabolic abnormalities play a pivotal role in the pathogenesis of urolithiasis, with distinct patterns observed in the NAME region. Hypercalciuria, driven by excessive dietary sodium, animal protein, and genetic predispositions, remains the most common lithogenic risk factor [55–58], particularly in NAME countries where processed food consumption is becoming more prevalent [59]. Similarly, hyperoxaluria due to high oxalate diets in the United Arab Emirates, Kuwait and Saudi Arabia—contributes to calcium oxalate stone formation [32,60], while hypocitraturia, exacerbated by low fruit/vegetable intake in Arab countries, further elevates risk [61,62]. The NAME region’s rising rates of obesity and type 2 diabetes introduce additional metabolic complexities; insulin resistance promotes urinary ammonium retention, creating a favorable environment for stone formation [63–66]. Collectively, these metabolic disturbances underscore the need for dietary counseling and biochemical screening in high-risk populations, especially where lifestyle transitions outpace public health interventions.

The infectious complications of urolithiasis represent a critical clinical concern in the NAME region, where specific epidemiological and healthcare factors amplify these risks. Obstructive stones, especially those causing ureteral blockage, lead to urinary stasis, fostering bacterial growth—particularly urease-producing gram-negative bacteria, as highlighted in regional research [67–69]. Environmental factors in NAME, such as high temperatures and dehydration, contribute to concentrated urine, while water scarcity in certain areas may further increase stone formation [48,70–73]. Additionally, the region’s rising diabetes epidemic (with a prevalence of 12.2%) heightens infection susceptibility due to insulin resistance [64,73–76]. There is also an observed upward trend in urinary tract infections, which may be partly associated with urolithiasis [77]. Compounding the issue, NAME faces high antimicrobial resistance rates, including methicillin-resistant *Staphylococcus aureus* (MRSA), complicating treatment [78,79]. These findings emphasize the necessity for enhanced clinical strategies, such as region-specific antibiotic prophylaxis and public health initiatives promoting early symptom detection. Addressing these infectious risks represents a pressing public health priority for NAME healthcare systems.

A critical methodological consideration emerges from the distinction between the NAME (GBD classification) and MENA (World Bank classification) regional definitions. As noted in our methods, the exclusion of Afghanistan – which demonstrated among the lowest burden rates in our study – from the MENA grouping could potentially lead to overestimation of urolithiasis burden when comparing NAME region data with MENA-based analyses.

The findings of this study have significant implications for public health and policy. First, the rising burden of urolithiasis calls for enhanced prevention efforts, including educational campaigns to raise awareness about risk factors such as dietary habits, fluid intake, and physical inactivity. Second, targeted interventions are needed to address the disparities in disease burden, particularly in low-SDI countries where underreporting and limited access to healthcare may mask the true extent of the problem. Investing in diagnostic infrastructure and training healthcare professionals in these settings could improve detection and management rates. Third, regional collaboration among NAME countries could facilitate the sharing of best practices and resources, such as adopting standardized protocols for urolithiasis management. Efforts to incorporate urolithiasis into broader non-communicable disease strategies, given its shared risk factors with conditions like obesity and diabetes, could also yield synergistic benefits.

Future research should focus on understanding the role of genetic predisposition and dietary patterns unique to the NAME region, which may influence urolithiasis risk. Additionally, exploring the cost-effectiveness of preventive measures, such as hydration campaigns and dietary counseling, could guide resource allocation. Longitudinal studies examining the impact of climate change on urolithiasis trends would also be valuable, given the region’s vulnerability to rising temperatures and water scarcity. Future iterations of GBD studies would benefit from subcategorizing kidney disease etiologies to better quantify stone-related morbidity burdens. Prospective regional studies tracking stone to end-stage renal disease progression could further clarify this relationship.

This study has several strengths that contribute to its significance and reliability. First, it utilizes data from the GBD 2021 study, one of the most comprehensive and up-to-date datasets available, ensuring robust and standardized estimates of health measures across 21 countries in the NAME region. Second, the study employs ASRs, allowing for accurate comparisons across populations with different demographic structures. Third, the analysis provides a nuanced understanding of the burden of urolithiasis by stratifying results by age, sex, and SDI, which highlights key variations and patterns across the region. However, this study has several limitations. The reliance on model-based estimates means that the results are subject to potential inaccuracies stemming from data gaps or biases in input data sources. For example, local variations in healthcare access, diagnostic practices, and reporting standards may not be fully captured. Also, while this study focuses on the NAME region, its findings may not be generalizable to other regions with different environmental and demographic profiles. Furthermore, this study did not explore the effects of potential risk factors that contribute to the urolithiasis burden, which needs further investigation. Finally, Our mortality estimates aggregate all urolithiasis-associated deaths without stratifying by specific causes. Future studies with cause-specific mortality data could refine these associations.

## Conclusion

The burden of urolithiasis in the NAME region has grown significantly over the past three decades, driven by rising incidence and prevalence rates. The condition remains a critical public health issue, particularly in middle-aged and older populations. While socioeconomic development associates with increased diagnosis and non-fatal burden, it does not necessarily mitigate the fatal outcomes of the disease. Policymakers and healthcare providers must prioritize awareness campaigns, early diagnosis, and prevention strategies tailored to regional needs. While improvements in socioeconomic conditions have led to enhanced healthcare infrastructure and increased disease detection, risk factors must also be controlled to ensure disease prevalence remains stable. Addressing modifiable risk factors such as dietary habits, hydration, and physical activity can substantially reduce the incidence of urolithiasis. Furthermore, enhancing access to specialized care and preventive services is crucial for managing the rising burden effectively. This study emphasizes the importance of regional analyses in understanding disease dynamics and guiding public health interventions.

## Supporting information

S1 File Underlying GBD 2021 data for all analyses. Complete dataset of urolithiasis burden measures (incidence, prevalence, deaths, YLDs, YLLs, DALYs) for 21 North Africa and Middle East countries, 1990–2021. Includes all-age numbers, age-standardized rates, percent change, and uncertainty intervals, stratified by sex, age, and country.
(CSV)

S2 File Percent change and temporal trends data. Percent change (1990–2021) with 95% uncertainty intervals for all burden measures, by country, sex, and age-standardized status. Used to generate Figure 1 and Table 1.
(CSV)

S3 File Temporal trends in all-age and age-standardized burden. Annual estimates (1990–2021) of all-age numbers and age-standardized rates for all burden measures in the NAME region, by sex. Used to generate Figure 1.
(CSV)

S4 File Age-standardized rates for map visualization. Age-standardized rates (ASRs) of incidence, prevalence, deaths, YLDs, YLLs, and DALYs for all 21 NAME countries, 1990 and 2021, by sex. Used to generate Figures 2 and 3.
(CSV)

S5 File Country rankings of age-standardized burden. Full ranking data for all burden measures across NAME countries, 1990 and 2021, including point estimates and 95% uncertainty intervals. Used to generate Figure 3.
(CSV)

S6 File Age-specific burden data, 1990 and 2021. Complete age-stratified rates (per 100,000) for incidence, prevalence, deaths, YLDs, YLLs, and DALYs in the NAME region, by sex and 5-year age groups. Used to generate Figure 4.
(CSV)

S7 File SDI-stratified burden data, 1990–2021. Country-year level data for deaths and DALYs with corresponding Socio-demographic Index (SDI) values. Used to generate Figure 5.
(CSV)
